# Potential Probes for Targeted Intraoperative Fluorescence Imaging in Gastric Cancer

**DOI:** 10.3390/cancers16244141

**Published:** 2024-12-12

**Authors:** Serena Martinelli, Laura Fortuna, Francesco Coratti, Federico Passagnoli, Amedeo Amedei, Fabio Cianchi

**Affiliations:** 1 Department of Clinical and Experimental Medicine, University of Florence, 50139 Florence, Italy; laura.fortuna@unifi.it (L.F.); corattif@gmail.com (F.C.); federico.passagnoli@unifi.it (F.P.); amedeo.amedei@unifi.it (A.A.); fabio.cianchi@unifi.it (F.C.); 2 Network of Immunity in Infection, Malignancy and Autoimmunity (NIIMA), Universal Scientific Education and Research Network (USERN), 50134 Florence, Italy

**Keywords:** gastric cancer, precision surgery, fluorescence

## Abstract

Gastric cancer (GC) is a malignant tumor of the gastrointestinal tract associated with high mortality rates and accounting for approximately 1 million new cases diagnosed annually. Surgery, particularly radical gastrectomy, remains the primary treatment; however, there are currently no specific approaches to better distinguish malignant from healthy tissue or to differentiate between metastatic and non-metastatic lymph nodes. As a result, surgeons have to remove all lymph nodes indiscriminately, increasing intraoperative risks for patients and prolonging hospital stay. Near-infrared fluorescence imaging with indocyanine green (ICG) can provide real-time visualization of the surgical field using both conventional laparoscopy and robotic mini-invasive precision surgery platforms. However, its application shows some limits, as ICG is a non-targeted contrast agent. Several studies are now investigating the potential efficacy of fluorescent targeted agents that could selectively bind to the tumor tissue, offering a valuable tool for metastatic mapping during robotic gastrectomy. This review aims to summarize the key fluorescent agents that have been developed to recognize GC markers, as well as those targeting the tumor microenvironment (TME) and metabolic features. These agents hold great potential as valuable tools for enhancing precision surgery in robotic gastrectomy procedures improving the clinical recovery of GC patients.

## Simple Summary

Gastric cancer (GC), a severe form of gastrointestinal malignancy, impacts around 1 million individuals each year and is associated with high mortality rates. Surgery, mainly radical gastrectomy, is the primary treatment, but challenges remain in distinguishing malignant from healthy tissue and identifying metastatic lymph nodes. Surgeons often remove all lymph nodes, increasing risks and recovery times. Near-infrared fluorescence imaging with indocyanine green (ICG) helps visualize surgical fields in minimally invasive procedures but is limited as a non-targeted contrast agent. Emerging targeted fluorescent agents aim to specifically bind to GC markers and the tumor microenvironment, facilitating metastatic mapping and removal precision in robotic gastrectomy. These innovations could improve surgical accuracy and improve recovery outcomes for GC patients.

## 1. Introduction

Gastric cancer (GC) ranks as the third leading cause of cancer-related deaths worldwide, with over 1 million new cases diagnosed each year [1,2,3]. Surgery remains the primary treatment for potentially curative outcomes in GC patients, making surgical quality a key research focus. However, current approaches for distinguishing malignant tissue from healthy mucosa (HM) largely rely on tactile and visual cues, as well as the surgeon’s expertise. Consequently, there is a risk of positive surgical margins (PSMs) or residual tumors remaining after resection [4]. Moreover, during surgery, it is impossible to discriminate between metastatic and non-metastatic lymph nodes, and this leads to the indiscriminate removal of all lymph nodes, increasing intraoperative risks for patients [5].

Over the past two decades, intraoperative navigation tools have been introduced across various aspects of oncosurgery to enhance care quality. Among these, near-infrared (NIR) fluorescence imaging with indocyanine green (ICG) has gained significant interest. This technology allows for real-time assessment of the operative field and anatomy. Conventional laparoscopy, as well as the da Vinci robotic platform, can integrate NIR fluorescence with ICG into the surgical process. NIR light is characterized by low absorption, low scattering, and low autofluorescence, enabling deeper tissue penetration than visible light. When ICG is used, specific structures such as lymphatic vessels, lymph nodes, and blood vessels can be clearly visualized [6,7,8], introducing new possibilities for intraoperative visualization of critical surgical parameters, such as lymphatic drainage, offering valuable decision support during surgery [9]. In addition, ICG helps ensure the completeness of lymph node removal, and improves surgical dissection and visualization of anastomotic perfusion. It also provides detailed information on the condition and composition of the tissue examined, as well as various perfusion parameters [10,11]. However, ICG accumulates passively in tumor tissue through the enhanced permeability and retention effect, yet it is not a targeted fluorescent contrast agent. Additionally, ICG’s use is limited because it loses its fluorescence after binding to proteins: indeed, ICG has a strong tendency to aggregate in the presence of proteins. This aggregation can disrupt its electronic structure, and this change can suppress the energy transitions required for fluorescence emission, effectively quenching the fluorescence [12].

There is a significant need for the development of a targeted fluorescent contrast agent that can actively accumulate in tumor tissue by recognizing specific biomarkers expressed by tumor cells. Such an agent would help to delineate the margins between tumor and surrounding normal tissues and allow for the precise visualization of metastatic lymph nodes during the surgery.

In this review, we describe the latest and promising fluorescent probes that have been developed to recognize GC markers, as well as those targeting the tumor microenvironment (TME) and tumor metabolic features. These molecules could be integrated into NIR technologies for use during gastrectomy.

## 2. Literature Search

PubMed was searched for articles in English using the following keywords, their associations and their acronyms: “gastric cancer”, “gastrectomy”, “surgery”, “biomarker”, “indocyanine green”, “near-infrared fluorescence”, “Da Vinci”, “robotic”, “clinical trials”, “mini invasive surgery”, “laparoscopy”, “dye”, “imaging”, “intraoperative”, “laparoscopy”, “microenvironment”, “microbiota”, and “intratumor”.

## 3. Gastrectomy with Fluorescence Imaging

Fluorescence imaging (FI) is currently the most popular approach used in image-guided surgery. It can be applied to conventional laparoscopy [13], as well as with mini-invasive robotic platforms, such as the da Vinci surgical system [14,15,16].

Traditional FI methods utilize fluorochromes within the visible light spectrum of 400 to 600 nm [17,18]. However, these methods are limited by a low penetration depth of just a few millimeters due to high light absorption by biological chromophores and significant background light/autofluorescence, resulting in a low signal-to-background ratio. To achieve high sensitivity and deeper penetration, wavelengths are ideally confined to the first NIR window, 650–900 nm, which offers reduced absorption, low autofluorescence, high spatial resolution, and high sensitivity [19]. Recently, significant advances in the second near-infrared region (NIR-II, 1000–1700 nm) are quickly developing, overcoming some limitations. NIR-II fluorescence imaging effectively addresses challenges such as intense tissue absorption, autofluorescence, and photon scattering, offering deep tissue penetration, micron-level spatial resolution, and high signal-to-background ratio [20].

**Figure 1:**
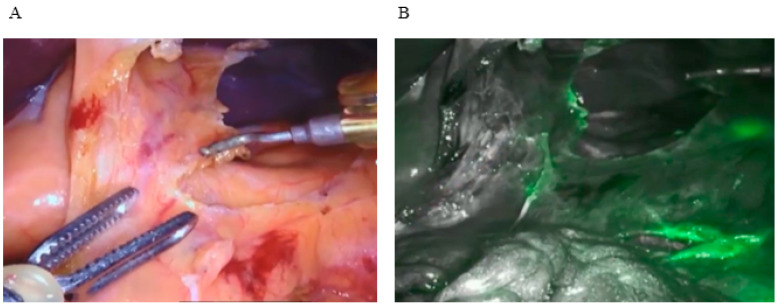
Lymph nodes identification in FireFly FI mode during Da Vinci gastrectomy using ICG. (**A**) White light view of gastric lymph nodes removal. (**B**) NIR view of the lymph nodes. A laser light source is used to excite the fluorophore at wavelengths around 800 nm, and the emitted light is captured by the image sensors on the endoscope.

Organic small molecular fluorophores, such as boron–dipyrromethene dyes, cyanine dyes, and their derivatives, are the primary sources for synthesizing current NIR fluorochromes. The Food and Drug Administration (FDA) approved ICG as an NIR dye for clinical use. ICG is a nontoxic agent and can be rapidly excreted from the human body, making a suitable tool for use in image-guided surgery. ICG is a water-soluble tricarbocyanin dye that rapidly binds to plasma proteins and is subsequently drained by the lymphatic system; it emits fluorescence at a wavelength of about 820 nm and it can be viewed by several devices. Current laparoscopic columns and some mini-invasive robotic platforms are equipped with a fluorescence visualization system during surgery. Specifically, the Da Vinci Xi robotic system has developed an innovative imaging technology for ICG visualization, featuring a laser source integrated into the robotic camera (Firefly), where surgeons can switch between white light, NIR light, and a composite vision [21]. The integration of NIR technology during gastrectomy procedures has shown promising results in improving tumor localization and surgical outcomes for GC patients, with a feasible and simple ICG administration to patients [22]. Studies have highlighted the significance of accurate tumor localization for establishing appropriate resection lines, impacting organ function preservation and curability, aiding in determining the resection line and ensuring negative margins, clearly visualizing lymph nodes, ultimately enhancing postoperative quality of life (Figure 1) [23,24,25,26,27,28].

Additionally, the use of NIR/ICG technology during gastrectomy for advanced gastric cancer (AGC) is being investigated in the iGreenGO study, aiming to evaluate its impact on surgical conduct and lymphadenectomy outcomes [29]. Moreover, a meta-analysis demonstrated increased lymph node retrieval, decreased operative time, and comparable complication rates, highlighting the efficacy and safety of this approach [27,30].

Several clinical trials have been performed and are ongoing to assess intraoperative fluorescence navigation using NIR/ICG during gastrectomy, which are summarized in Table 1.

Despite its several advantages, ICG passively accumulates in tumor tissue through the enhanced permeability and retention effect, but it remains a non-targeted fluorescent contrast agent. Furthermore, the application of ICG in molecular imaging probes is constrained because it loses its fluorescence upon protein binding because of the ICG–proteins aggregation that suppress the energy transitions required for fluorescence emission [12]. There is a strong need for the development of a targeted fluorescent contrast agent that can actively accumulate in tumor tissue by recognizing a specific biomarker expressed by tumor cells. Such an agent could distinguish the margins between tumors and surrounding normal tissues and enable the visualization of metastatic lymph nodes during gastrectomy.

## 4. Probes Targeting Gastric Cancer Surface Markers

Current studies on GC lymph node dissection using NIR revealed that surgical navigation with non-tumor-specific fluorescence can increase the total number of detected lymph nodes and ensure completion of dissection but does not improve accuracy. Achieving accurate lymph node dissection for GC depends on tumor-specific tracking of positive lymph nodes. Although tumor-specific tracers are developing rapidly and related clinical studies are emerging, there are still few specific reports on lymph node metastasis, indicating that lymph node tracking remains a difficult problem to solve [32].

Combining molecular probes with specific tumor biomarkers allows the probes to finely target cancer cells. This targeting strategy in molecular imaging can greatly enhance the accuracy and sensitivity of image-guided surgery. This approach involves conjugating molecular probes with cancer-targeting ligands, such as small molecules, peptides, proteins, antibodies, and aptamers [33,34,35].

The first use in humans of tumor-specific intraoperative FI for real-time surgical visualization of cancer was performed in ovarian cancer by targeting the folate-α receptor, which is overexpressed in 90–95% of epithelial ovarian cancers [36]. Moreover, Cao and colleagues developed a new peptide that can specifically target human epidermal growth factor receptor 2 (HER2) conjugated to the NIR-ICG to obtain a probe with high and fast binding affinity and no observable toxicity to cells and tissues. Tumor resection was successfully performed under the guidance of the novel molecule in the subcutaneous SKOV3 mice model [37].

Regarding HER2 as a GC biomarker, Trastuzumab conjugated with IRDye800CW demonstrated high affinity for HER2, allowing for clear visualization of HER2-positive tumors in animal models: the fluorescence signal peaked at 24 h post-injection, enabling the effective differentiation of tumors from surrounding tissues during surgery [38].

Previous basic research suggested that integrins play a crucial molecular role in GC lymph node metastasis [39]. In particular, the integrin alpha(α)v beta(β)3 receptor is frequently implicated in the progression of malignant tumors and is associated with cancer angiogenesis and metastasis [40]. The αvβ3 receptor is abundantly expressed in proliferating tumor cells and activated endothelial cells, but it is expressed at very low levels in healthy endothelial cells, quiescent vascular cells, and other cells, making it a suitable target for tracers or anticancer agents [41,42]. The Arg-Gly-Asp (RGD) sequence binds to the integrin αvβ3 with high affinity and strong selectivity; hence, these polypeptides can mark lesions and angiogenesis for tumor detection, showing great potential for tumor diagnosis and treatment. Radiolabeled RGD peptides and their analogs, especially 99mTc3PRGD2 molecule, have been extensively studied for use in the non-invasive imaging of integrin αvβ3 receptor expression in diverse cancer types including lung cancer [43], breast cancer [44], and esophageal cancer [45,46], and provided effective lymph node imaging in those cancers, and the derivative 99mTc-oncoFAPI PET-CT showed promising results for lymph node imaging in GC [47].

Therefore, a very recent clinical trial aims to explore the potential application of 99mTc3PRGD2 and other probes in the molecular imaging of GC, guiding lymph node dissection and tracing, and accumulating preliminary clinical data to develop corresponding fluorescent probes for intraoperative tracing (NCT06435741). Cheng and colleagues investigated peritoneal carcinomatosis from GC in a mouse model by using a self-developed surgical navigation system combining optical molecular imaging with an RGD-ICG probe, achieving a sensitivity and specificity of up to 93.93% and 100%, respectively, with a diagnostic index (DI) of 193.93% and diagnostic accuracy rate of 93.93% [48].

**Figure 2:**
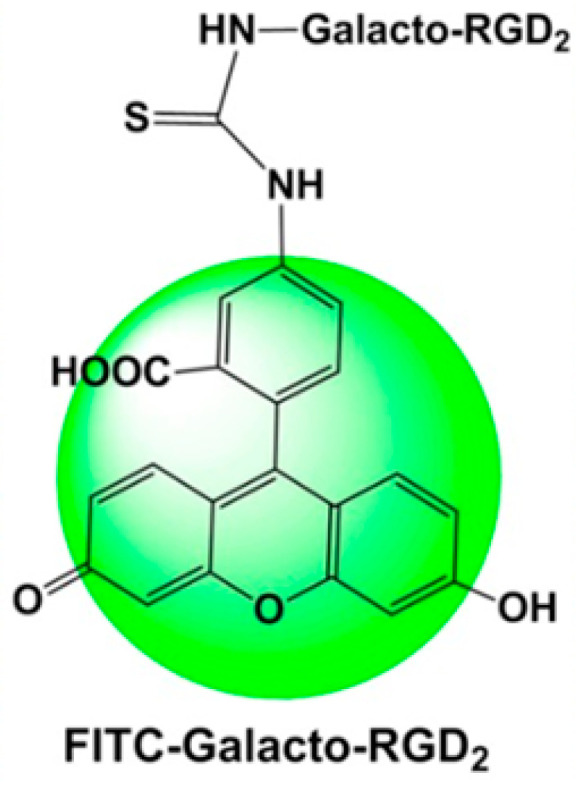
Molecular structure of the fluorescent probes based on FITC-conjugated cyclic RGD peptides (FITC-RGD2, FITC-3P-RGD2, and FITC-Galacto-RGD2). Adapted from Zheng et al. [49].

Zheng and colleagues highlighted the efficacy of FITC-conjugated cyclic RGD peptides, specifically FITC-Galacto-RGD2 and FITC-3P-RGD2, to stain integrin αvβ3/αvβ5 in human carcinoma tissues, including GC, showing a strong correlation between the fluorescent intensity and the integrin expression levels [49]. Indeed, the study found that GC tissues exhibited minimal staining when using FITC-Galacto-RGD2, due to the low expression levels of integrin αvβ3/αvβ5 in these tissues, indicating that the fluorescent probe may not be effective for all types of cancer, mainly those with lower integrin expression (Figure 2) [49].

The human carcinoembryonic antigen (CEA), also known as CEACAM5, is an attractive target due to its well-defined nature as a tumor antigen. It is minimally present during human embryonic development, absent in normal adult tissues, and highly expressed in various solid gastrointestinal cancers. Several groups have developed chimeric antibodies conjugated with fluorescent agents in order to target human pancreatic cancer in orthotopic xenograft mouse models [50,51,52,53], as well as colorectal cancer in mice models and patients [54,55,56,57]. Regarding GC, although many biomarkers have been reported, CEACAM5 is the most frequently used in clinical practice and shows promising potential for the development of possible fluorescent molecules against this target [58,59,60,61,62]. The SGM-101 anti-CEACAM5 antibody was conjugated to a near-infrared (NIR) fluorophore to facilitate CEA-targeted fluorescence image-guided surgery at an absorbance band centered at 705 nm [63]. The molecule achieved tumor area accumulation in the GC xenograft mouse model with SGM-101, suggesting that tumor-specific NIR imaging may be a feasible tool for image-guided surgery [64]. A promising clinical trial using SGM-101 is ongoing on patients with peritoneal carcinomatosis from CEA-overexpressing digestive cancer (FLUOCAR-1, NCT02784028). SGM-101 was also assessed in humans for intraoperative detection of CRC: in a pilot study (NCT02973672), 38 patients with CRC were administered the molecule, and the authors observed a good tolerability, safety, and efficacy of the drug [65]. Recently, Cox and colleagues evaluated the ability of a humanized anti-CEA antibody (M5A) previously developed by Yazaki [66] and then conjugated it with an 800 nm NIR dye to target GC in orthotopic mouse models, achieving a high tumor-to-background ratio [67,68]. Yazaki et al. developed a prototype anti-CEA-swPEG-IR800 conjugate with improved blood circulation half-life and tumor sensitivity, enhancing optical imaging for intraoperative visualization of CEA-expressing GI cancers [69]. In a very recent work, two patient-derived GC lines were developed from surgical samples of two patients undergoing gastrectomy for adenocarcinoma. Tumor fragments obtained from these samples were implanted into the mice stomach to create patient-derived orthotopic xenograft (PDOX) models [70].This approach seems promising as a clinical tool for detecting the extent of the disease to determine resectability and for visualizing tumor margins during GC resection.

EpCAM (epithelial cell adhesion molecule) has also emerged as a promising target for epithelial-derived tumors, due to their EpCAM overexpression, exploiting it for NIR fluorescence-guided surgery, improving the precision of tumor resections in various types of cancer. The anti-EpCAM monoclonal antibody conjugated with IRDye800CW effectively delineate tumors in colorectal, breast, and head and neck cancers, allowing for the detection of millimeter-sized nodules invisible to the naked eye [71]. Houvast and colleagues recently designed two ankyrin repeat proteins (DARPins) targeting EpCAM that have been validated for their ability to provide clear tumor delineation in preclinical models, indicating their potential for pan-carcinoma visualization [72]. The study, primarily examining colon cancer, established that a dosage of 6 nmol and an imaging time point of 24 h post-injection were optimal for both the two DARPins, and it found a good tumor-to-background ratio [72]. In addition, the anti-EpCAM antibody fragment, EpCAM-F800, has shown high specificity and rapid accumulation in CRC and breast cancer [73]. Although the potential use of EpCAM as a multi-tumor target is promising, including epithelial-derived GC, challenges remain in optimizing the specificity and safety of these agents for clinical applications.

Likewise, ICAM1 (Intercellular Adhesion Molecule 1) is a transmembrane glycoprotein that plays a crucial role in immune responses and cellular signaling, facilitating the adhesion of leukocytes to endothelial cells during the inflammatory response [74]. Under physiological conditions, ICAM-1 is expressed at low levels on the surface of endothelial cells, epithelial cells, and some immune cells, and in inflammatory diseases, such as cancer, it is overexpressed, revealing to be a potential biomarker for early GC diagnosis and prognosis, suggesting its role in precision surgery for the patients [75]. It was found that 49% of the patients showed ICAM-1 expression through immunohistochemical staining. The rate of ICAM-1 expression was higher in advanced stages of cancer, especially in cases with lymph node metastasis and liver metastasis [76]. Indeed, Wang et al. designed a novel antibody–drug conjugate (ADC) targeting chromosomally unstable GC (CINGC) using ICAM1 as a molecular target, offering a potential therapeutic approach for this aggressive subtype of GC [77].

Olfactomedin 4 (OLFM4), an antiapoptotic factor glycoprotein that facilitates cell adhesion, has been shown to be upregulated in various types of cancer and involved in many cellular processes such as cell adhesion, apoptosis, and cell proliferation [78]. In GC, the clinicopathological relevance of OLFM4 expression has been reported to be involved not only in early stages of GC but also as a useful prognostic marker for advanced GC, which is encouraging for further studies exploring OLFM4 as a potential target for GC therapy or tracking [79]. OLFM4 is also linked to lymph node metastasis in GC patients, and its depletion inhibits GC cell tumorigenicity in vitro and in vivo by enhancing caspase-3-dependent apoptosis [80,81,82]. Due to its role in GC progression and prognosis, OLFM4 can be a potential biomarker for precision surgery, aligning with the era of precision medicine advancements [83].

Claudin 18.2 (CLDN18.2), member of a family of proteins which are the most relevant components of the tight junctions, has also been identified as a potential target for precision surgery in AGC. A molecular imaging strategy using an antibody specific for CLDN18.2, 5C9 has been investigated to detect specific lesions and guide surgery [84]. This strategy involves the synthesis of imaging probes such as 124I-5C9 and Cy5.5-5C9, which have demonstrated specificity for CLDN18.2 in cellular experiments and have been used in immuno-PET and FI for tumor delineation and visualization. In addition, an NIR-II fluorescent probe, FD1080-5C9, was designed to facilitate the complete surgical removal of CLDN18.2-positive lesions, demonstrating the potential for precise surgical guidance in AGC and other tumors [84].

Among others, aberrant glycosylation of proteins and lipids is considered a hallmark of cancer [85,86]. During oncogenesis, immature mucin-type O-glycans and fucosylated glycan antigens, such as sialyl-Lewis^a^ (sLe^a^/CA19.9), are overexpressed on the cell membrane of tumor cells, providing opportunities for molecular imaging. Houvast and colleagues demonstrated the efficacy of real-time glycan-based imaging of gastrointestinal tumors using the CH88.2 antibody conjugated to the NIR fluorophore IRDye800CW. Specific binding of the antibody was confirmed on human gastrointestinal tissues and various gastrointestinal cell lines. The tracer specificity was further validated in vivo using subcutaneous mouse models of gastrointestinal tumors. By combining a chimeric antibody with clinical-grade NIR imaging systems, this approach could facilitate rapid clinical translation, not only for this tracer but also for the broader concept of cancer imaging using glycan-targeted tracers [87].

The epithelial-to-mesenchymal transition (EMT) is widely acknowledged as a key pathway through which cancer cells acquire metastatic potential and is strongly associated with poor patient survival [88,89]. While EMT plays a critical role in fetal development, its occurrence in cancer cells is an early indicator of metastasis. Research has shown that the cell surface protein CD146 serves as a distinct marker for EMT activation in cancer cells [90]. Owing to its varying expression in metastases and advanced primary tumors, along with its minimal presence in normal tissue, CD146 has gained significant attention as a potential target for early cancer diagnosis, prognosis, and treatment. Wang and colleagues developed a new fluorescent antibody anti-CD146 and conjugated it to a superparamagnetic iron oxide nanoparticle (SPION) and to an NIR fluorescent agent. The authors injected the molecule through the tail vein of mice to target the xenograft model of MKN45 GC cells, demonstrating that the tumor could be clearly visualized as early as 30 min after injection. Peak tumor uptake was quantified 24 h after injection [91].

Since cholecystokinin 2 receptor (CCK2R) is overexpressed in lung, colon, thyroid, pancreatic, and GC, but is largely absent in most non-tumor tissues [92], Wayua and colleagues developed an NIR ligand that targets CCK2R-positive tumors. The authors performed the synthesis and biological evaluation of LS-288, a CCK2R-targeted NIR conjugate. The results documented that CRL-LS288 selectively bound to CCK2R-positive tumor cells with high affinity and preferentially localized to CCK2R-expressing HEK293 murine tumor xenografts, also revealing the presence of distant tumor metastases [93]. These findings suggest the application of image-guided surgery in GC tumors.

Recently, the combination of NIR FI and magnetic resonance imaging (MRI) has been successfully applied for precise and efficient in vivo tumor monitoring [94,95]. Therefore, Yang and colleagues developed hollow nanocomposites for dual-modal MRI/FLI imaging to improve imaging accuracy for both GC in situ and metastases. The nanocomposites were then amino-modified and cross-linked with Cy7.5 and with folic acid. The study results demonstrated that the nanocomposite possessed strong targeting abilities and showed excellent biosafety both in vitro and in vivo. In an orthotopic metastatic GC model using nude mice, the authors successfully achieved precise monitoring of primary and metastatic tumors through MRI and FI guided by bioluminescence imaging for tumor localization [96]. Table 2 synthesizes the information described in this paragraph for each probe.

Overall, this targeting approach paves the way for potential applications in patients for better, more radical cytoreductive surgery. Further research is essential to refine these approaches and validate their efficacy in different patient populations.

## 5. Probes Targeting Tumor Microenvironment Biomarkers of Gastric Cancer

As is well known, any type of tumor is strongly characterized by its own microenvironment that shapes its characteristics and aggressiveness, supporting proliferation and invasion into surrounding tissues [97,98,99]. For this reason, in addition to probes that bind tumor biomarkers, there are many studies demonstrating the efficacy of probes that target specific features of the tumor microenvironment, such as neo-angiogenesis capacity or metalloprotease production.

Tumor blood vessels carry specific markers that are usually related to angiogenesis. Some markers were found to be expressed only in tumor-activated endothelial cells, making them suitable targets for tumor-tracing [100].

GEBP11 is a peptide that specifically binds to the tumor vasculature of human GC. In vivo tracking experiments have shown that the GEBP11 isotope probe effectively targets GC tissue with a specific distribution, suggesting its potential for clinical applications [101]. This innovative probe had relevant potential applications in GC diagnosis, vascular targeting therapy, and evaluation of therapeutic effects, indicating its classification in biomedical technology and cancer treatment [101]. In addition, the development of an NIR dye-labeled GEBP11 dimer peptide targeted to the tumor vasculature holds promise for image-guided surgery in GC. Moreover, the GEBP11 peptide was successfully conjugated with Cy5.5, enhancing tumor-specific binding and detection capabilities [102]. Furthermore, the use of the GEBP11 peptide in a dual-modality imaging probe allowed visualization of tumor angiogenesis in GC models, highlighting its potential for in vivo imaging guidance during surgery, holding great promise for improving the accuracy and efficacy of image-guided surgery in GC [103]. About GEBP11 and GC imaging, a new magnetic resonance and fluorescence (MR/Fluo) dual-modality imaging probe is developed by covalently coupling 2,3-dimercaptosuccinnic acid-coated paramagnetic nanoparticles (DMSA-MNPs) and Cy5.5 to the GEBP11 peptide, showing good imaging properties, high stability, and low cytotoxicity [104].

Bevacizumab-IRDye800CW is emerging as a promising tool to improve tumor detection in CRC via fluorescence-guided surgery. This approach leverages the targeting of vascular endothelial growth factor α (VEGFα), which is frequently overexpressed in tumors, allowing for improved visualization during surgical procedures. Studies suggest that this fluorescent tracer can significantly improve the tumor-to-background ratio, enhancing the surgeon’s ability to distinguish between cancerous and healthy tissues, not only in CRC but also in breast cancer, showing to be a promising candidate for GC as well [105,106,107].

Ogawa and colleagues examined whether matrix metalloprotease-14 (MMP-14) was a candidate enzyme in FI for the diagnosis of peritoneal metastasis in GC [108]. MMP-14 showed significantly higher expression in cancerous tissues compared to normal tissues, and its protease activity can be exploited to convert the fluorescent probe BODIPY-MMP into its active form. This specificity enhanced the effectiveness of intraoperative FI, assisting surgeons in precisely identifying metastatic sites [108].

In addition to MMPs, other proteases can be used as probes activators, such as cysteine cathepsins, members of the papain-like cysteine protease family. The 6QC-ICG probe is a fluorescently quenched substrate that is activated in the presence of cysteine cathepsins, which are particularly abundant in tumors. The probe is initially non-fluorescent due to the quenching mechanism. When the probe encounters the proteases, they cleave the substrate at specific amide bonds, releasing a fragment that contains the fluorescent reporter (ICG) [109].

Fibroblast activation protein-alpha (FAPα) plays a crucial role in most solid cancer types, including GC. FAPα is overexpressed by cancer-associated fibroblasts (CAFs), which are a major component of the tumor stroma and have been reported to exhibit increased glycolysis. This metabolic shift enables CAFs to produce high-energy nutrients, such as lactate, pyruvate, and other metabolites, which are then transferred to malignant cells. These nutrients support the energetic and biosynthetic needs of cancer cells, facilitating their growth, proliferation, and survival [110,111,112]. Recent advancements in imaging techniques leaded to the development of an FAP-targeted NIR fluorescent dye FTL-S-S0456, which provide high specificity and sensitivity for detecting solid tumors, including GC with high specificity and affinity, producing high-contrast, long-lasting images of malignant lesions with little or no retention in healthy tissue [113].

Fibronectin (FN) levels are significantly elevated in CAFs compared to normal fibroblasts, making it a prime target for imaging agents [114,115]. The development of dual-modal MR/NIRF imaging contrast agents targeting FN represents a significant advance in the non-invasive diagnosis of GC and peritoneal metastases [114]. Moreover, the dual-modal agents like CREKA-Cy7-(Gd-DOTA) and FeGdNP-ICG/GOx-RGD2-mPEG demonstrated high specificity and sensitivity in detecting GC and peritoneal metastasis [116]. These agents exploit the fibronectin upregulation in CAFs, enhancing imaging capabilities and potentially improving clinical outcomes.

A recent study identified multiple biomarkers and created a fluorescent nanomachine for the combined diagnosis of GC, offering a novel design for a functionalized DNA nanomachine and a practical approach to translating serum biomarkers into clinical diagnostic tools. In detail, miR-5585-5p and PLS3 mRNA were identified through next-generation sequencing and RT-qPCR as biomarkers, enabling highly sensitive and specific early gastric cancer (EGC) screening [117].

Very recently, a nanocomposite fluorescence probe, ICG@MSNs-PEG-Ab, has been developed by coupling an anti-PD-L1 antibody to mesoporous silica nanoparticles (MSNs) loaded with ICG and coated with polyethylene glycol (PEG) for breast cancer. Hopefully, this kind of probe could also be applied to GC in the future. This nanocomposite probe exhibited first-rate biocompatibility, emitting stable fluorescence in the NIR-II spectrum, with enhanced resistance to photodegradation [118].

Quantum dots (QDs), a subtype of nanoparticles, possess properties that make them suitable as NIR fluorescent probes, offering an alternative to organic dyes. Typically composed of inorganic semiconducting materials, they are generally less than 50 nm in size and exhibit high photostability for prolonged imaging, an easier multicolor imaging, and exceptional brightness for detecting receptor-level concentrations [119,120,121]. In the NIR range, QDs outperform organic dyes in photoluminescence quantum yields (PLQYs), reaching up to 45% in aqueous media. Their longer lifetimes in the excited state allow delayed microscopy, reducing cellular autofluorescence. In addition, their high surface area/volume ratio facilitates efficient functionalization, allowing multimodal probes for imaging at different tissue depths. NIR QDs, in particular, offer advantages such as small size (down to 30 nm), biocompatibility, and high photostability, making them often superior to conventional dyes. Together with their ability to carry targeting motifs and therapeutic agents, QDs hold great promise for the advancement of preclinical biomedical therapeutics and imaging [122,123]. Leveraging these properties of QDs, a fluorescent probe was designed to specifically label GC cells in vitro. Primary QDs were conjugated with the tumor-associated glycoprotein 72 (TAG-72) monoclonal antibody CC49, resulting in CC49-QDs probe, which is capable of targeting tumor cells with high specificity. After evaluating the diameter and emission spectrum of the CC49-QDs, they were successfully applied in immunofluorescence analysis [124,125]. Moreover, NIR QD probes able to detect simultaneously biomarkers such as cytokeratin 20 (CK20) and proliferating cell nuclear antigen (PCNA) in GC tissues were synthesized, revealing stronger immunostaining ability compared to visible QDs [125,126]. Recently, Zhang and colleagues developed a probe leveraging perovskite quantum dots (PQDs) and peptide ligands. Using CsPbBr3 PQDs modified with azithromycin (AZI) and a specific polypeptide ligand targeting CD44v6, a gastric cancer biomarker, they created the AZI-PQDs probe. This perovskite-based probe is capable of specifically identifying gastric cancer tumors [127] (Table 3).

## 6. Metabolic GC Tracers

Tumor cells are characterized by increased glucose uptake compared to normal cells, a phenomenon known as the Warburg effect [128]. As a result, glucose consumption is significantly elevated in cancer cells. Monitoring cellular glucose consumption is therefore an effective method to distinguish cancerous from non-cancerous tissues. This approach is exemplified by the common clinical use of 2-deoxy-2-(18F) fluoro-D-glucose (18F–FDG), a radiolabeled glucose analog. 18F–FDG is routinely used in positron emission tomography (PET) to image tumors and their metastases in vivo [129,130].

**Figure 3:**
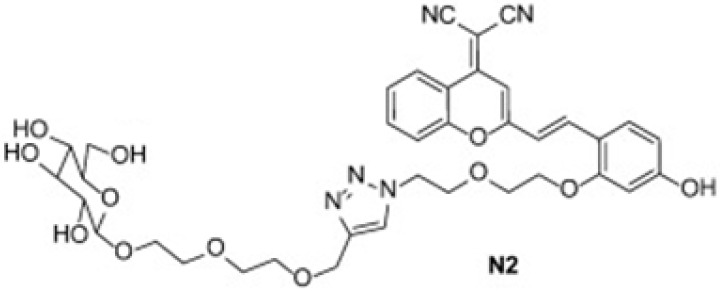
Molecular structure of the glucose analog N2. Adapted from Fang et al. [137].

The new synthesized NIR fluorescent dye-labeled glucose analogs can be used in cancer cell imaging. The first NIR fluorescent dye-labeled glucose analog was Pyro-2DG, a derivative of 2-deoxyglucose (2-DG), effective for cancer detection and photodynamic therapy [131]. Later, Cy5.5-2DG was used for tumor imaging in mice, but its uptake was not inhibited by D-glucose, raising concerns about its glucose transporter (GLUT) delivery [132]. IRDye800CW 2-DG showed good cancer cell uptake and was blockable by excess 2-DG or D-glucose, but its negative charge hindered cell membrane permeability [133]. CyNE 2-DG had better cell permeability and higher fluorescence but was not tested in animal models [134]. Other analogs like Cypate-2DG and ICG-Der-02-2DG showed tumor-targeting abilities with different clearance rates [135]. A novel NIR dye, DCPO, with boosted photostability [136], inspired the synthesis of five glucose analogs by Fang and colleagues, among which the N2 molecule showed potential for sensing glucose uptake in cancer cells through GLUT-1 glucose transporters, which are overexpressed in a wide variety of solid tumors. N2 could be used to monitor cellular glucose consumption and, therefore, could be applied in the bioimaging of cancer cells (Figure 3) [137].

Zhao and colleagues investigated the tumor-targeting capability of the NIR fluorescent heptamethine carbocyanine dye, MHI-148, in cultured GC cells as well as in GC cell-derived and patient-derived tumor xenograft (PDX) models. The results of the study demonstrated that the dye selectively accumulated in tumor areas in both xenograft models. The authors demonstrated that hypoxia enhanced the uptake of MHI-148 in GC cells primarily through the activation of hypoxia inducible factor 1 α (HIF1α), which upregulated organic-anion-transporting polypeptide (OATP) transporters, leading to increased dye accumulation in tumor regions [138]. In addition to MHI-148, another cyanine dye, IR-783, was taken up by cancer cells through the OATP pathway and showed localization primarily in the mitochondria and lysosomes, suggesting that the dyes may interact with specific organelles [139]. This mechanism highlights the potential of using hypoxia-targeted imaging agents in clinical settings.

**Figure 4:**
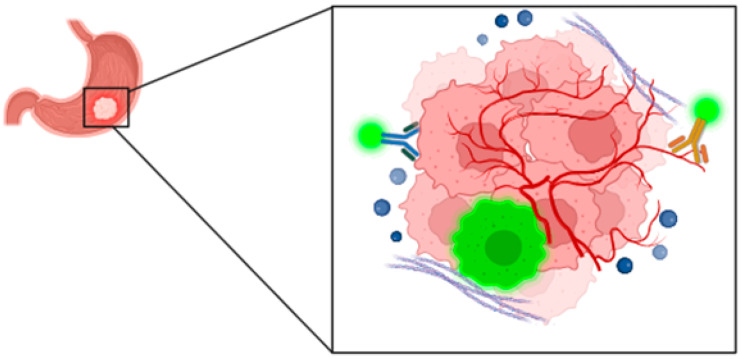
Schematic representation of the three types of probes described in the previous sections: in blue, we show an example of a probe targeting tumor cells. In yellow, we show a probe that targets the tumor microenvironment, in this case, the blood vessels. Metabolic tracers are represented by a fluorescent probe internalized by tumor cells.

Another metabolic characteristic of solid tumors is the acid microenvironment, due to increased metabolic rates, higher glycolysis, and inadequate vasculature [140,141]. The acidic microenvironment can be exploited to activate specific probes such as ONM-100, which was designed with an ultra-pH-sensitive amphiphilic polymer that responds to the low pH levels found in the tumor microenvironment. This sensitivity allowed the nanoprobe to remain inactive in neutral or alkaline conditions, but when the pH drops due to tumor acidosis, the polymer irreversibly dissociated, leading to the release of the ICG fluorescent dye, revealing a strong fluorescence signal that can be detected during surgery [142]. A similar mechanism was utilized by another probe that focused on DNA nanoassemblies that responded to pH changes. The DNA nanoassembly detected GC cells by recognizing specific biomarkers prevalent in these cells, ensuring stability in normal tissues while being activated in the acidic tumor microenvironment [143] (Table 4). A limitation of this approach may be that other inflammatory conditions may also have an acidic pH, resulting in the activation of the probe outside the tumor site. A schematic representation of three kind of probes described is summarized in Figure 4.

## 7. The Intratumor Microbiota as Biomarker

Next-generation sequencing has shed light on the intricate relationship between GC, microbial profiles, and precision surgery. Studies have revealed distinct genetic alterations in GC patients of different origins, underscoring the potential of precision medicine to address oncological disparities [144,145]. The gastric microbiota, especially lactic acid bacteria and oral microflora, play a critical role in gastric carcinogenesis by inducing chronic inflammation and promoting the production of nitroso compounds [146,147,148,149]. In addition, the identification of specific microbial markers, such as Streptococcus, Pseudomonas, Fusobacterium, Selenomonas, Peptostreptococcus, and Prevotella, has shown promise in distinguishing GC from non-cancerous patients, offering new avenues for non-invasive diagnostic approaches and potential therapeutic targets [150,151]. The GC microbiota may be a signature of cancer development and progression, characterized by distinct microbial profiles identified in different molecular subtypes [144,152]. Research indicates that *Helicobacter pylori* and other organisms in the intratumoral microbiome have a significant impact on GC pathogenesis and progression [144,153]. Studies have shown that GC is associated with decreased microbial diversity, with a specific enrichment of bacteria such as Helicobacter, Lactobacillus, Streptococcus, Prevotella, and Bacteroides in tumor samples compared with nonmalignant tissue [144,153]. Furthermore, the presence of GC is linked to a specific fungal mycobiomic signature, characterized by altered fungal composition and ecology, indicating a potential role of the fungal microbiome in the pathogenesis of GC [154].

Identifying a distinct microbial signature within GC tissue could pave the way for the development of specialized probes that target specific microbial species associated with the tumor. These probes could enhance surgery precision by improving the localization of tumor metastasis, allowing for more accurate identification of cancerous regions. This approach holds potential to complement existing techniques and increase the overall effectiveness of fluorescence-guided surgery in GC treatment.

## 8. Discussion

Intraoperative fluorescence-targeted imaging probes in GC represent a transformative approach to improve surgical outcomes through tumor visualization and margin delineation. Current research focuses on the development of probes with high specificity, stability, and biocompatibility. Among these, NIR fluorophores, peptide-based probes, and QDs stand out for their excellent optical properties and ability to be conjugated with tumor-specific ligands such as antibodies or peptides targeting biomarkers.

Regarding the advantages and disadvantages of TME-targeted probes compared to surface marker-targeted probes, we need to argue some points. TME-targeted probes have broad applicability; they can identify common features such as hypoxia and angiogenesis, which are shared by many cancer types, including GC. In addition, these biomarkers are often distributed throughout the tumor, allowing for better visualization of poorly accessible regions within the tumor mass, providing insights into tumor physiology and interactions with surrounding tissues, offering insights into aggressiveness and progression. These probes have great potential to be effective in heterogeneous tumors; in fact, tumor microenvironment features tend to be more consistent within a tumor than surface markers, making them suitable for imaging heterogeneous tumors. On the other hand, TME features may also be present in inflamed or damaged non-cancerous tissues, reducing the specificity for GC. In addition, probes targeting TME may fail to distinguish between tumor types, as many tumors share similar microenvironmental characteristics. For probes targeting surface markers, a major advantage is high specificity because binding to antigens or receptors is unique to GC cells, allowing precise tumor identification and delineation, distinguishing tumor cells from surrounding healthy tissue, decreasing unnecessary removal of non-cancerous tissue during surgery, and may help identify and resect metastatic lymph nodes more effectively. On the other hand, these kinds of probes may not reach deeper tumor regions due to heterogeneity and limited vascular accessibility. Moreover, some GCs exhibit low expression of surface markers, reducing probe effectiveness and potentially missing tumor regions. Additionally, there is the possibility that tumor markers may change over time due to mutations or treatment, which can reduce probe reliability. An optimal approach may be to combine both types of probes to obtain comprehensive imaging and effective treatment planning.

Regarding the toxicity risk of the probes, certain nanoparticles can accumulate within the body, potentially causing long-term toxicity. Additionally, materials designed to enhance biocompatibility may, in rare cases, provoke immune responses or hypersensitivity reactions. The effects are often dose-dependent, as high concentrations of NIR probes can overwhelm the body’s elimination processes, resulting in localized or systemic toxicity. Furthermore, the breakdown of NIR probes could release harmful byproducts, depending on the chemical stability of their components. Innovations in probe formulation, such as biodegradable or self-assembling materials, could mitigate toxicity concerns. Finally, incorporating artificial intelligence and machine learning into image analysis may optimize real-time intraoperative decision-making.

To address these challenges, comprehensive preclinical studies are urgently needed to assess the safety of NIR probes, including their pharmacokinetics, biodistribution, and long-term effects.

## 9. Conclusions

Despite promising results, the above-mentioned strategies often lack tumor applicability due to intratumor heterogeneity and diversity of expressed markers. This challenge is mostly pronounced when patients receive neoadjuvant treatment prior to surgery. Such treatment often results in tumors that assume an irregular shape and lose their structural integrity, complicating the ability of probes to effectively target and penetrate cancerous tissue. Altered tissue architecture and variable marker expression can hinder the probes’ accuracy and efficacy, making precise tumor localization difficult.

Another limitation of fluorescence-guided precision surgery for GC is that the gastric acidic pH could affect the effectiveness of the probes in reaching the tumor site. The harsh acidic environment may degrade or alter the stability and function of the fluorescent probes, decreasing their ability to target and highlight the cancerous tissue accurately, thereby compromising the precision of the surgery.

Extensive studies involving large patient populations will be essential to validate the efficacy of these probes before they can be considered for clinical use. Such research is crucial to ensure that the probes consistently deliver accurate and reliable results across diverse patient groups, addressing any variability in tumor characteristics and treatment responses. Only with comprehensive data and successful validation can these probes be confidently integrated into clinical practice. Once fully validated, these advancements could lead to more accurate tumor localization, reduce damage to healthy tissue, and consequently decrease patient discomfort. Moreover, by enhancing surgical precision, targeted tracers may also improve long-term outcomes, including faster recovery, shorter hospital stay, decreased complication rates, a lower likelihood of tumor recurrence, and an overall better prognosis for GC patients.

## Figures and Tables

**Table 1:** List of the clinical trials on robotic gastrectomy with the use of ICG. Available at https://www.clinicaltrials.gov/.

Clinical Trial Number	Name of the Study	Status	Phase of the Study	Type of Study
NCT05229874	Effect of CNSI vs. ICG in Lymph Node Tracing During Gastrectomy (FUTURE-01)	Active, not recruiting	Phase 2	Interventional
NCT01926743	Identification of Complete Lymph Node Removal by Application of Near Infrared Fluorescence Imaging in Laparoscopic and Robotic Gastrectomy	Completed	Not Applicable	Interventional
NCT04734821	Fluorescence Image Guided Foregut Surgery (FOREGUT)	Recruiting	Not Applicable	Interventional
NCT04107623	Quantitative Fluorescent Guided Robotic Surgery for Cancer of the Gastroesophageal Junction	Completed	Not Applicable	Interventional
NCT03931044	Fluorescence Image-Guided Lymphadenectomy in Robotic Gastrectomy (IG-MIG)	Unknown	Not Applicable	Interventional
NCT03396354	Prospective Comparison of Surgical Outcomes with Using Integrated Robotic Technology Versus Conventional Laparoscopy for Gastric Cancer Surgery	Unknown	Not Applicable	Interventional
NCT04943484	ICG (Indocyanine Green) Imaging Fluorescence Technology in Surgical Treatment of Advanced Gastric Cancer (iGreenGO)	Recruiting	Not Applicable	Observational
NCT04352894	Intraoperative ICG Fluorescence Imaging for Peritoneal Carcinomatosis Detection	Unknown	Not Applicable	Interventional
NCT05685862	Laser Speckle Contrast Imaging, Surgical Eye & ICG Fluorescence Imaging for Perfusion Assessment of the Gastric Conduit (CONDOR-I)	Recruiting	Not Applicable	Observational
NCT05369117	Application of Indocyanine Green Labeled Fluorescent Laparoscopy in Proximal Gastric Cancer	Not yet recruiting	Not Applicable	Interventional
NCT05687617	Near-infrared Imaging with Indocyanine Green for Detection of Peritoneal Metastases for Gastric Adenocarcinoma.	Terminated	Phase 2	Interventional
NCT04056260	ICG-NIR Guided Lymph Node Dissection in Gastric Cancer	Unknown	Not Applicable	Interventional
NCT05618821	Clinical Outcomes of Indocyanine Green Tracer Using in Laparoscopic Gastrectomy with Lymph Node Dissection for Remnant Gastric Cancer	Recruiting	Phase 2	Interventional
NCT01441310	Laparoscopic Sentinel Node Navigation Surgery for Gastric Cancer (SNNS)	Completed	Phase 2	Interventional
NCT04352894	Intraoperative ICG Fluorescence Imaging for Peritoneal Carcinomatosis Detection	Unknown	Not Applicable	Interventional
NCT05720598	Staging LaParoscopy to Assess Lymph NOde InvoLvement in Advanced GAstric Cancer (POLA)	Recruiting	Not Applicable	Interventional
NCT04591028	A Study to Evaluate Indocyanine Green Lymphangiography to Improve Lymphadenectomy in Gastric Cancer Patients	Withdrawn	Phase 4	Interventional
NCT04593615	Indocyanine Green Tracer Using in Laparoscopic Radical Gastrectomy for Locally Advanced Gastric Cancer (CLASS-11)	Active, not recruiting	Not Applicable	Interventional
NCT04973475	Indocyanine Green Tracer Using in Laparoscopic Distal Gastrectomy for Early Gastric Cancer	Not yet recruiting	Phase 2	Interventional
NCT04611997	IGG Using in Laparoscopic Gastrectomy for Locally Advanced Gastric Cancer After Neoadjuvant Chemotherapy	Active, not recruiting	Phase 3	Interventional
NCT03050879	Indocyanine Green Tracer Using in Laparoscopic Gastrectomy with Lymph Node Dissection (ICGTinLG) [31]	Completed	Phase 2	Interventional
NCT06421220	Evaluation of the Efficacy and Safety of Indocyanine Green Tracing in 3D Fluorescent Laparoscopic Lymph Node Dissection for Gastric Cancer	Not yet recruiting	Not Applicable	Interventional

**Table 2:** Summary of the parameters highlighting fluorescence properties, performance metrics, working mechanisms, and influencing factors for probe effectiveness.

Probe/Target	Fluorescence Properties	Performance	Working Principles	Factors Affecting Sensitivity/Specificity
Folate-α Receptor Targeting	Excitation: ~700 nm Emission: ~800 nm	High affinity for ovarian cancers	Conjugation with NIR dye for targeting folate-α receptor overexpressed in ovarian cancer.	High receptor expression enhances specificity; low expression in some tumors reduces sensitivity.
HER2 Targeting (Trastuzumab + IRDye800CW)	Excitation: ~774 nm Emission: ~789 nm	Peak signal at 24 h post-injection; specific tumor visualization	Antibody conjugation targeting HER2 for enhanced visualization during surgery.	HER2 overexpression improves sensitivity; variable expression across tumors affects performance.
Integrin αvβ3 Targeting (RGD Peptides)	Excitation: ~800 nm Emission: ~820 nm	Sensitivity: 93.93%; Specificity: 100%	Binds integrin αvβ3 overexpressed in tumor angiogenesis and metastasis; enables molecular imaging and surgery.	Low integrin expression decreases sensitivity; specificity maintained by low expression in healthy tissues.
CEACAM5 Targeting (SGM-101)	Excitation: ~705 nm Emission: ~720 nm	Strong tumor accumulation; good tolerability	NIR-labeled antibody targets CEACAM5, overexpressed in gastrointestinal cancers, for real-time surgical guidance.	CEACAM5 overexpression improves detection; heterogeneous expression impacts specificity.
EpCAM Targeting (IR-Dye800CW)	Excitation: ~774 nm Emission: ~789 nm	Clear tumor delineation; mm-sized nodules detectable	Antibody/DARPins conjugated to NIR dyes target EpCAM for precise imaging in epithelial-derived tumors.	Tumor overexpression of EpCAM increases sensitivity; systemic overexpression may reduce specificity.
ICAM1 Targeting	Excitation: ~700–800 nm Emission: ~800–850 nm	High expression in metastatic GC cases	Antibody–drug conjugates (ADCs) targeting ICAM1 in advanced GC for therapeutic and imaging purposes.	High ICAM1 expression in advanced cases enhances specificity; low expression in early GC limits sensitivity.
OLFM4 Targeting	Excitation: ~700 nm Emission: ~800 nm	Promising prognostic marker; lymph node metastasis tracking	Targets OLFM4 glycoprotein involved in cell adhesion and apoptosis, associated with GC progression.	Overexpression in advanced GC improves sensitivity; low expression in early stages reduces utility.
CLDN18.2 Targeting	Excitation: ~650 nm Emission: ~670 nm	Demonstrates tumor specificity in imaging	Antibody-based NIR imaging (e.g., Cy5.5-5C9) enables precise lesion delineation in gastric adenocarcinoma (AGC).	Specific CLDN18.2 expression increases accuracy; reduced signal in negative tissues limits sensitivity.
Aberrant Glycosylation Targeting	Excitation: ~800 nm Emission: ~820 nm	Effective lymph node imaging; high tracer specificity	CH88.2 antibody targets glycan-based markers for real-time imaging of gastrointestinal tumors.	Overexpression of glycan markers enhances sensitivity; variability across cancer subtypes may limit application.
CD146 Targeting	Excitation: ~700 nm Emission: ~800 nm	High tumor–background ratio; early visualization	Antibody conjugated with NIR and nanoparticles targets EMT marker CD146, linked to GC metastases.	High CD146 expression in metastatic cells increases sensitivity; variability impacts specificity.
CCK2R Targeting	Excitation: ~700 nm Emission: ~800 nm	High affinity for CCK2R-positive tumors	NIR ligand selectively binds CCK2R, overexpressed in various cancers, including GC.	High receptor density improves detection; limited receptor presence in some tumors reduces sensitivity.
Dual-Modal Imaging (MRI + FI)	Excitation: ~750–800 nm Emission: ~820 nm	Improved localization of primary/metastatic tumors	Nanocomposites with Cy7.5 and folic acid provide MRI and NIR imaging capabilities for precise tumor tracking.	Strong targeting enhances specificity; non-specific uptake in normal tissues may reduce signal-to-noise ratio.

**Table 3:** Summary of the fluorescence properties, sensitivity/specificity, working principles, and factors influencing probe performance in GC microenvironment imaging.

Probe/Target	Fluorescence Properties	Performance	Working Principles	Factors Affecting Sensitivity/Specificity
GEBP11-Cy5.5	Excitation: ~650 nm Emission: ~670 nm	High tumor-specific binding , clinical potential in image-guided surgery	Targets tumor vasculature, binds to tumor-specific markers on endothelial cells.	Tumor-specific vasculature expression enhances performance. Non-specific binding or systemic degradation reduces effectiveness.
Bevacizumab-IRDye800CW	Excitation: ~774 nm Emission: ~794 nm	Improved tumor-to-background ratio, enhanced visibility in surgery	Binds VEGFα, overexpressed in tumors, enabling fluorescence-guided surgery.	VEGFα expression level in tumor microenvironment affects targeting. Systemic uptake decreases specificity.
BODIPY-MMP	Excitation: ~500–520 nm Emission: ~520–540 nm	High specificity in detecting metastatic GC tissues	Activated by MMP-14 protease activity, leading to fluorescence.	High MMP-14 levels improve detection. Low enzyme activity in certain tumor regions reduces effectiveness.
6QC-ICG	Excitation: ~780 nm Emission: ~805 nm	Effective in detecting cysteine cathepsins in tumor environment	Activated by cysteine cathepsins, releasing fluorescent ICG reporter.	High cathepsin levels increase sensitivity. Overlapping fluorescence signals or enzyme-independent activation may reduce specificity.
FTL-S-S0456	Excitation: ~700 nm Emission: ~750 nm	High specificity and sensitivity for solid tumors, long-lasting imaging	Targets FAPα on cancer-associated fibroblasts in tumor stroma.	Overexpression of FAPα enhances performance. Limited FAPα expression in some tumors reduces effectiveness.
CREKA-Cy7-(Gd-DOTA)	Excitation: ~720 nm Emission: ~740 nm	High sensitivity and specificity in detecting fibronectin in GC	Targets fibronectin in CAFs; dual-modal MR/NIRF imaging.	Elevated fibronectin levels improve detection. Poor probe stability or retention in non-target tissues reduces performance.
CC49-QDs	Excitation: ~450–500 nm Emission: ~650–800 nm (NIR range)	Highly specific labeling of tumor cells	Conjugates QDs with anti-TAG-72 monoclonal antibodies to target tumor cells.	Tumor-associated glycoprotein 72 (TAG-72) expression enhances specificity. Cross-reactivity with other glycoproteins may reduce accuracy.
AZI-PQDs	Excitation: ~450–490 nm Emission: ~520–550 nm	Specific identification of gastric cancer tumors	Uses CsPbBr3 PQDs modified with azithromycin and peptide ligand targeting CD44v6, a GC biomarker.	Strong CD44v6 expression improves specificity. Aggregation or degradation of PQDs may lower sensitivity.
ICG@MSNs-PEG-Ab	ICG@MSNs-PEG-Ab	High stability and low cytotoxicity, excellent biocompatibility	Combines mesoporous silica nanoparticles loaded with ICG, PEG coating, and anti-PD-L1 antibody for NIR-II imaging.	High expression of PD-L1 enhances targeting. Photodegradation or systemic inflammation can decrease specificity.
Dual-Marker QDs (CK20/PCNA)	Excitation: ~450–500 nm Emission: ~650–800 nm (NIR range)	Strong immunostaining ability, higher performance than visible QDs	Targets cytokeratin 20 (CK20) and proliferating cell nuclear antigen (PCNA) for simultaneous biomarker detection.	Dual targeting improves specificity. Poor functionalization of QDs or overlapping signals may affect accuracy.

**Table 4:** We cover fluorescence properties, sensitivity, specificity, and factors affecting probe performance in detecting tumor-specific metabolic features such as glucose uptake and acidic microenvironments.

Probe/Target	Fluorescence Properties	Performance	Working Principles	Factors Affecting Sensitivity/Specificity
Pyro-2DG	Excitation: ~600 nm Emission: ~650 nm	Effective for cancer detection and photodynamic therapy	NIR fluorescent derivative of 2-deoxyglucose; targets high glucose consumption in tumors.	Strong GLUT expression and Warburg effect improve performance. Non-specific uptake reduces specificity.
Cy5.5-2DG	Excitation: ~675 nm Emission: ~694 nm	Moderate uptake in cancer cells, but not GLUT-specific	Tumor targeting through glucose metabolism, but non-blockable by excess glucose, raising concerns about mechanism.	Non-specific uptake reduces specificity. Proper conjugation enhances targeting.
IRDye800CW 2-DG	Excitation: ~774 nm Emission: ~794 nm	Good cancer cell uptake, blockable by 2-DG or D-glucose; limited by poor membrane permeability	NIR fluorescent glucose analog targeting GLUT transporters; negatively charged dye limits penetration.	Improved permeability enhances specificity. Negative charge limits efficiency.
CyNE 2-DG	Excitation: ~710 nm Emission: ~740 nm	High fluorescence; not tested in animal models	NIR glucose analog with better membrane permeability for cancer imaging.	Increased permeability and proper GLUT targeting improve sensitivity. Lack of in vivo validation may limit confidence in specificity.
Cypate-2DG	Excitation: ~785 nm Emission: ~810 nm	Tumor-targeting ability with varied clearance rates	NIR glucose analog exploiting high glucose uptake in tumors.	Effective clearance and strong GLUT targeting enhance performance. Poor metabolic stability reduces specificity.
DCPO-N2	Excitation: ~720 nm Emission: ~750 nm	Effective for sensing glucose uptake in cancer cells via GLUT-1 transporters	NIR dye with high photostability; selectively monitors glucose consumption in tumors.	High GLUT-1 expression improves sensitivity. Insufficient probe targeting specificity decreases performance.
MHI-148	Excitation: ~710–740 nm Emission: ~750–780 nm	Selective tumor accumulation in hypoxic regions via OATPs; tested in xenograft models	Heptamethine cyanine dye; hypoxia activates HIF1α, enhancing OATPs expression and dye uptake in tumors.	High hypoxia levels and HIF1α activation improve sensitivity. Reduced hypoxia or insufficient OATP expression limits specificity.
IR-783	Excitation: ~710 nm Emission: ~780 nm	Preferential localization in mitochondria and lysosomes; taken up via OATPs	Cyanine dye targeting cancer cells through OATPs; localizes in specific organelles like mitochondria and lysosomes.	Strong OATP expression enhances sensitivity. Off-target accumulation or improper organelle targeting decreases specificity.
ONM-100	Excitation: ~800 nm Emission: ~820 nm	High specificity for acidic tumor microenvironment	Ultra-pH-sensitive amphiphilic polymer; remains inactive in neutral/alkaline pH but releases ICG dye in acidic tumor environments.	Tumor acidosis improves specificity. Variations in tumor pH or non-specific activation reduce performance.
DNA Nanoassembly Probe	Excitation: depends on specific fluorescent markers Emission: varies by dye	High specificity and stability in normal tissues; activated in acidic tumor environments	DNA nanoassemblies respond to pH changes; specifically recognizes GC biomarkers and activates in acidic microenvironments.	Acidic tumor pH and robust biomarker targeting enhance specificity. Inconsistent activation or off-target pH sensitivity may reduce performance.

## References

[1] Smyth E.C., Nilsson M., Grabsch H.I., van Grieken N.C., Lordick F. (2020). Gastric cancer. Lancet.

[2] Chhikara B.S., Parang K. (2023). Global Cancer Statistics 2022: The trends projection analysis. Chem. Biol. Lett..

[3] Bray F., Laversanne M., Sung H., Ferlay J., Siegel R.L., Soerjomataram I., Jemal A. (2024). Global cancer statistics 2022: GLOBOCAN estimates of incidence and mortality worldwide for 36 cancers in 185 countries. CA Cancer J. Clin..

[4] Tringale K.R., Pang J., Nguyen Q.T. (2018). Image-guided surgery in cancer: A strategy to reduce incidence of positive surgical margins. Wiley Interdiscip. Rev. Syst. Biol. Med..

[5] Ke B., Liang H. (2021). Current status of lymph node dissection in gastric cancer. Chin. J. Cancer Res..

[6] Schaafsma B.E., Mieog J.S., Hutteman M., van der Vorst J.R., Kuppen P.J., Löwik C.W., Frangioni J.V., van de Velde C.J., Vahrmeijer A.L. (2011). The clinical use of indocyanine green as a near-infrared fluorescent contrast agent for image-guided oncologic surgery. J. Surg. Oncol..

[7] Sakamoto E., Dias A.R., Ramos M.F.K.P., Safatle-Ribeiro A.V., Zilberstein B., Ribeiro Junior U. (2021). Indocyanine green and near-infrared fluorescence imaging in gastric cancer precision surgical approach. Arq. Gastroenterol..

[8] Sherwinter D.A., Boni L., Bouvet M., Ferri L., Hyung W.J., Ishizawa T., Kaleya R.N., Kelly K., Kokudo N., Lanzarini E. (2022). Use of fluorescence imaging and indocyanine green for sentinel node mapping during gastric cancer surgery: Results of an intercontinental Delphi survey. Surgery.

[9] Chalopin C., Pfahl A., Köhler H., Knospe L., Maktabi M., Unger M., Jansen-Winkeln B., Thieme R., Moulla Y., Mehdorn M. (2023). Alternative intraoperative optical imaging modalities for fluorescence angiography in gastrointestinal surgery: Spectral imaging and imaging photoplethysmography. Minim. Invasive Ther. Allied Technol..

[10] Knospe L., Gockel I., Jansen-Winkeln B., Thieme R., Niebisch S., Moulla Y., Stelzner S., Lyros O., Diana M., Marescaux J. (2022). New Intraoperative Imaging Tools and Image-Guided Surgery in Gastric Cancer Surgery. Diagnostics.

[11] Kashchenko V.A., Lodygin A.V., Krasnoselsky K.Y., Zaytsev V.V., Kamshilin A.A. (2023). Intra-abdominal laparoscopic assessment of organs perfusion using imaging photoplethysmography. Surg. Endosc..

[12] Ogawa M., Kosaka N., Choyke P.L., Kobayashi H. (2009). In vivo molecular imaging of cancer with a quenching near-infrared fluorescent probe using conjugates of monoclonal antibodies and indocyanine green. Cancer Res..

[13] Chen Q.-Y., Zhong Q., Liu Z.-Y., Li P., Lin G.-T., Zheng Q.-L., Wang J.-B., Lin J.-X., Lu J., Cao L.-L. (2023). Indocyanine green fluorescence imaging-guided versus conventional laparoscopic lymphadenectomy for gastric cancer: Long-term outcomes of a phase 3 randomised clinical trial. Nat. Commun..

[14] DiMaio S., Hanuschik M., Kreaden U., Rosen J., Hannaford B., Satava R.M. (2011). The da Vinci Surgical System. Surgical Robotics: Systems Applications and Visions.

[15] de Jongh C., Cianchi F., Kinoshita T., Kingma F., Piccoli M., Dubecz A., Kouwenhoven E., van Det M., Mala T., Coratti A. (2024). Surgical Techniques and Related Perioperative Outcomes After Robot-assisted Minimally Invasive Gastrectomy (RAMIG): Results From the Prospective Multicenter International Ugira Gastric Registry. Ann. Surg..

[16] Choi S., Kim N.Y., Kim Y.N., Park S.H., Kim K.Y., Cho M., Kim Y.M., Hyung W.J., Kim H.I. (2023). Fluorescence-guided Two-port Robotic Gastrectomy Versus Conventional Laparoscopic Gastrectomy: A Nonrandomized Controlled Trial. Ann. Surg. Open.

[17] Nagaya T., Nakamura Y.A., Choyke P.L., Kobayashi H. (2017). Fluorescence-Guided Surgery. Front. Oncol..

[18] Lauwerends L.J., van Driel P., Baatenburg de Jong R.J., Hardillo J.A.U., Koljenovic S., Puppels G., Mezzanotte L., Löwik C., Rosenthal E.L., Vahrmeijer A.L. (2021). Real-time fluorescence imaging in intraoperative decision making for cancer surgery. Lancet Oncol..

[19] Li S., Cheng D., He L., Yuan L. (2021). Recent Progresses in NIR-I/II Fluorescence Imaging for Surgical Navigation. Front. Bioeng. Biotechnol..

[20] Chen Y., Xue L., Zhu Q., Feng Y., Wu M. (2021). Recent Advances in Second Near-Infrared Region (NIR-II) Fluorophores and Biomedical Applications. Front. Chem..

[21] Zhu B., Sevick-Muraca E.M. (2015). A review of performance of near-infrared fluorescence imaging devices used in clinical studies. Br. J. Radiol..

[22] Barbato G., Cammelli F., Braccini G., Staderini F., Cianchi F., Coratti F. (2022). Fluorescent lymphography for thoracic duct identification: Initial experience of a simplified and feasible ICG administration. Int. J. Med. Robot..

[23] Takahashi J., Yoshida M., Ohdaira H., Nakaseko Y., Nakashima K., Kamada T., Suzuki N., Sato T., Suzuki Y. (2023). Efficacy and Safety of Gastrointestinal Tumour Site Marking with da Vinci-Compatible Near-Infrared Fluorescent Clips: A Case Series. World J. Surg..

[24] Sagawa H., Saito M., Ito S., Hayakawa S., Ueno S., Okubo T., Tanaka T., Ogawa R., Takahashi H., Matsuo Y. (2022). Near infrared ray-guided surgery using Firefly technology of the daVinci Xi system and intraoperative upper gastrointestinal endoscopy for subtotal gastrectomy and surgery for cancer of the gastroesophageal junction. BMC Surg..

[25] Deng H.Y., Luo J., Li S.X., Li G., Alai G., Wang Y., Liu L.X., Lin Y.D. (2019). Does robot-assisted minimally invasive esophagectomy really have the advantage of lymphadenectomy over video-assisted minimally invasive esophagectomy in treating esophageal squamous cell carcinoma? A propensity score-matched analysis based on short-term outcomes. Dis. Esophagus.

[26] Liu J., Ruan H., Zhao K., Wang G., Li M., Jiang Z. (2014). Comparative study on da Vince robotic and laparoscopic radical gastrectomy for gastric cancer. Zhonghua Wei Chang Wai Ke Za Zhi.

[27] Abate M., Drebin H., Shimada S., Fei T., McKinley S., Poruk K., Ferguson B., Neuwirth M., Tang L.H., Vardhana S. (2024). Feasibility and Efficacy of Sentinel Lymph Node Mapping in Gastric Cancer. Ann. Surg. Oncol..

[28] Senent-Boza A., García-Fernández N., Alarcón-Del Agua I., Socas-Macías M., de Jesús-Gil Á., Morales-Conde S. (2024). Impact of tumor stage and neoadjuvant chemotherapy in fluorescence-guided lymphadenectomy during laparoscopic gastrectomy for gastric cancer: A propensity score-matched study in a western center. Surgery.

[29] Lombardi P.M., Mazzola M., Nicastro V., Giacopuzzi S., Baiocchi G.L., Castoro C., Rosati R., Fumagalli Romario U., Bonavina L., Staderini F. (2022). The iGreenGO Study: The Clinical Role of Indocyanine Green Imaging Fluorescence in Modifying the Surgeon’s Conduct During the Surgical Treatment of Advanced Gastric Cancer-Study Protocol for an International Multicenter Prospective Study. Front. Oncol..

[30] Zhang Z., Deng C., Guo Z., Liu Y., Qi H., Li X. (2023). Safety and efficacy of indocyanine green near-infrared fluorescent imaging-guided lymph node dissection during robotic gastrectomy for gastric cancer: A systematic review and meta-analysis. Minim. Invasive Ther. Allied Technol..

[31] Chen Q.Y., Xie J.W., Zhong Q., Wang J.B., Lin J.X., Lu J., Cao L.L., Lin M., Tu R.H., Huang Z.N. (2020). Safety and Efficacy of Indocyanine Green Tracer-Guided Lymph Node Dissection During Laparoscopic Radical Gastrectomy in Patients with Gastric Cancer: A Randomized Clinical Trial. JAMA Surg..

[32] Achterberg F.B., Deken M.M., Meijer R.P.J., Mieog J.S.D., Burggraaf J., van de Velde C.J.H., Swijnenburg R.J., Vahrmeijer A.L. (2021). Clinical translation and implementation of optical imaging agents for precision image-guided cancer surgery. Eur. J. Nucl. Med. Mol. Imaging.

[33] Barth C.W., Gibbs S.L. Fluorescence Image-Guided Surgery—A Perspective on Contrast Agent Development. Proceedings of the SPIE—The International Society for Optical Engineering.

[34] Wang H., Li X., Tse B.W., Yang H., Thorling C.A., Liu Y., Touraud M., Chouane J.B., Liu X., Roberts M.S. (2018). Indocyanine green-incorporating nanoparticles for cancer theranostics. Theranostics.

[35] Ishizawa F.D.D.T., Kokudo N., Rosenthal R.J. (2015). Fluorescence Imaging for Surgeons.

[36] van Dam G.M., Themelis G., Crane L.M., Harlaar N.J., Pleijhuis R.G., Kelder W., Sarantopoulos A., de Jong J.S., Arts H.J., van der Zee A.G. (2011). Intraoperative tumor-specific fluorescence imaging in ovarian cancer by folate receptor-α targeting: First in-human results. Nat. Med..

[37] Cao R., Li R., Shi H., Liu H., Cheng Z. (2023). Novel HER2-Targeted Peptide for NIR-II Imaging of Tumor. Mol. Pharm..

[38] Jeong K., Kong S.H., Bae S.W., Park C.R., Berlth F., Shin J.H., Lee Y.S., Youn H., Koo E., Suh Y.S. (2021). Evaluation of Near-infrared Fluorescence-conjugated Peptides for Visualization of Human Epidermal Receptor 2-overexpressed Gastric Cancer. J. Gastric. Cancer.

[39] Jin H., Varner J. (2004). Integrins: Roles in cancer development and as treatment targets. Br. J. Cancer.

[40] Eliceiri B.P., Cheresh D.A. (1999). The role of alphav integrins during angiogenesis: Insights into potential mechanisms of action and clinical development. J. Clin. Investig..

[41] Niu G., Chen X. (2011). Why integrin as a primary target for imaging and therapy. Theranostics.

[42] Desgrosellier J.S., Cheresh D.A. (2010). Integrins in cancer: Biological implications and therapeutic opportunities. Nat. Rev. Cancer.

[43] Zhu Z., Miao W., Li Q., Dai H., Ma Q., Wang F., Yang A., Jia B., Jing X., Liu S. (2012). 99mTc-3PRGD2 for integrin receptor imaging of lung cancer: A multicenter study. J. Nucl. Med..

[44] Chen Z., Fu F., Li F., Zhu Z., Yang Y., Chen X., Jia B., Zheng S., Huang C., Miao W. (2018). Comparison of [(99m)Tc]3PRGD(2) Imaging and [(18)F]FDG PET/CT in Breast Cancer and Expression of Integrin α(v)β(3) in Breast Cancer Vascular Endothelial Cells. Mol. Imaging Biol..

[45] Zheng S., Chen Z., Huang C., Chen Y., Miao W. (2019). [99mTc]3PRGD2 for integrin receptor imaging of esophageal cancer: A comparative study with [18F]FDG PET/CT. Ann. Nucl. Med..

[46] Lv N., Gao S., Bai L., Ji B., Xue J., Ge X., Chen B. (2019). Advantages of (99m)Tc-3PRGD(2) SPECT over CT in the preoperative assessment of lymph node metastasis in patients with esophageal cancer. Ann. Nucl. Med..

[47] Jia X., Li X., Jia B., Yang Y., Wang Y., Liu Y., Ji T., Xie X., Yao Y., Qiu G. (2023). The role of [(99m)Tc]Tc-HFAPi SPECT/CT in patients with malignancies of digestive system: First clinical experience. Eur. J. Nucl. Med. Mol. Imaging.

[48] Cheng H., Chi C., Shang W., Rengaowa S., Cui J., Ye J., Jiang S., Mao Y., Zeng C., Huo H. (2017). Precise integrin-targeting near-infrared imaging-guided surgical method increases surgical qualification of peritoneal carcinomatosis from gastric cancer in mice. Oncotarget.

[49] Zheng Y., Ji S., Czerwinski A., Valenzuela F., Pennington M., Liu S. (2014). FITC-conjugated cyclic RGD peptides as fluorescent probes for staining integrin αvβ3/αvβ5 in tumor tissues. Bioconjug. Chem..

[50] Lwin T.M., Murakami T., Miyake K., Yazaki P.J., Shivley J.E., Hoffman R.M., Bouvet M. (2018). Tumor-Specific Labeling of Pancreatic Cancer Using a Humanized Anti-CEA Antibody Conjugated to a Near-Infrared Fluorophore. Ann. Surg. Oncol..

[51] Maawy A.A., Hiroshima Y., Kaushal S., Luiken G.A., Hoffman R.M., Bouvet M. (2013). Comparison of a chimeric anti-carcinoembryonic antigen antibody conjugated with visible or near-infrared fluorescent dyes for imaging pancreatic cancer in orthotopic nude mouse models. J. Biomed. Opt..

[52] Metildi C.A., Kaushal S., Pu M., Messer K.A., Luiken G.A., Moossa A.R., Hoffman R.M., Bouvet M. (2014). Fluorescence-guided surgery with a fluorophore-conjugated antibody to carcinoembryonic antigen (CEA), that highlights the tumor, improves surgical resection and increases survival in orthotopic mouse models of human pancreatic cancer. Ann. Surg. Oncol..

[53] Tran Cao H.S., Kaushal S., Metildi C.A., Menen R.S., Lee C., Snyder C.S., Messer K., Pu M., Luiken G.A., Talamini M.A. (2012). Tumor-specific fluorescence antibody imaging enables accurate staging laparoscopy in an orthotopic model of pancreatic cancer. Hepatogastroenterology.

[54] Kaushal S., McElroy M.K., Luiken G.A., Talamini M.A., Moossa A.R., Hoffman R.M., Bouvet M. (2008). Fluorophore-conjugated anti-CEA antibody for the intraoperative imaging of pancreatic and colorectal cancer. J. Gastrointest. Surg..

[55] Folli S., Wagnières G., Pèlegrin A., Calmes J.M., Braichotte D., Buchegger F., Chalandon Y., Hardman N., Heusser C., Givel J.C. (1992). Immunophotodiagnosis of colon carcinomas in patients injected with fluoresceinated chimeric antibodies against carcinoembryonic antigen. Proc. Natl. Acad. Sci. USA.

[56] Metildi C.A., Kaushal S., Luiken G.A., Talamini M.A., Hoffman R.M., Bouvet M. (2014). Fluorescently labeled chimeric anti-CEA antibody improves detection and resection of human colon cancer in a patient-derived orthotopic xenograft (PDOX) nude mouse model. J. Surg. Oncol..

[57] Keller R., Winde G., Terpe H.J., Foerster E.C., Domschke W. (2002). Fluorescence endoscopy using a fluorescein-labeled monoclonal antibody against carcinoembryonic antigen in patients with colorectal carcinoma and adenoma. Endoscopy.

[58] Liu J.N., Wang H.B., Zhou C.C., Hu S.Y. (2014). CEACAM5 has different expression patterns in gastric non-neoplastic and neoplastic lesions and cytoplasmic staining is a marker for evaluation of tumor progression in gastric adenocarcinoma. Pathol. Res. Pract..

[59] Matsuoka T., Yashiro M. (2018). Biomarkers of gastric cancer: Current topics and future perspective. World J. Gastroenterol..

[60] Toh J., Hoppe M.M., Thakur T., Yang H., Tan K.T., Pang B., Ho S., Roy R., Ho K.Y., Yeoh K.G. (2020). Profiling of gastric cancer cell-surface markers to achieve tumour-normal discrimination. BMJ Open Gastroenterol..

[61] Lin J.-P., Lin J.-X., Ma Y.-B., Xie J.-W., Yan S., Wang J.-B., Lu J., Chen Q.-Y., Ma X.-F., Cao L.-L. (2020). Prognostic significance of pre- and post-operative tumour markers for patients with gastric cancer. Br. J. Cancer.

[62] Feng F., Tian Y., Xu G., Liu Z., Liu S., Zheng G., Guo M., Lian X., Fan D., Zhang H. (2017). Diagnostic and prognostic value of CEA, CA19–9, AFP and CA125 for early gastric cancer. BMC Cancer.

[63] Gutowski M., Framery B., Boonstra M.C., Garambois V., Quenet F., Dumas K., Scherninski F., Cailler F., Vahrmeijer A.L., Pèlegrin A. (2017). SGM-101: An innovative near-infrared dye-antibody conjugate that targets CEA for fluorescence-guided surgery. Surg. Oncol..

[64] Koo E., Jeong K.-Y., Yoo J., Shin J.-Y., Lim L., Kim H.-M., Park J.-Y., Lee Y.-S., Framery B., Dumas K. (2022). NIR fluorochrome labeled anti-carcinoembryonic antigen monoclonal antibody for gastric cancer-specific image guided surgery. J. Clin. Oncol..

[65] Boogerd L.S.F., Hoogstins C.E.S., Schaap D.P., Kusters M., Handgraaf H.J.M., van der Valk M.J.M., Hilling D.E., Holman F.A., Peeters K., Mieog J.S.D. (2018). Safety and effectiveness of SGM-101, a fluorescent antibody targeting carcinoembryonic antigen, for intraoperative detection of colorectal cancer: A dose-escalation pilot study. Lancet Gastroenterol. Hepatol..

[66] Yazaki P.J., Sherman M.A., Shively J.E., Ikle D., Williams L.E., Wong J.Y., Colcher D., Wu A.M., Raubitschek A.A. (2004). Humanization of the anti-CEA T84.66 antibody based on crystal structure data. Protein Eng. Des. Sel..

[67] Cox K.E., Turner M.A., Amirfakhri S., Lwin T.M., Hosseini M., Ghosh P., Obonyo M., Murakami T., Hoffman R.M., Yazaki P.J. (2024). Humanized Anti-Carcinoembryonic Antigen Antibodies Brightly Target and Label Gastric Cancer in Orthotopic Mouse Models. J. Surg. Res..

[68] DeLong J.C., Murakami T., Yazaki P.J., Hoffman R.M., Bouvet M. (2017). Near-infrared-conjugated humanized anti-carcinoembryonic antigen antibody targets colon cancer in an orthotopic nude-mouse model. J. Surg. Res..

[69] Yazaki P., Lwin T., Minnix M., Li L., Sherman A., Molnar J., Miller A., Frankel P., Chea J., Poku E. (2019). Improved antibody-guided surgery with a near-infrared dye on a pegylated linker for CEA-positive tumors. J. Biomed. Opt..

[70] Cox K.E., Turner M.A., Lwin T.M., Amirfakhri S., Kelly K.J., Hosseini M., Ghosh P., Obonyo M., Hoffman R.M., Yazaki P.J. (2024). Targeting Patient-Derived Orthotopic Gastric Cancers with a Fluorescent Humanized Anti-CEA Antibody. Ann. Surg. Oncol..

[71] van Driel P.B., Boonstra M.C., Prevoo H.A., van de Giessen M., Snoeks T.J., Tummers Q.R., Keereweer S., Cordfunke R.A., Fish A., van Eendenburg J.D. (2016). EpCAM as multi-tumour target for near-infrared fluorescence guided surgery. BMC Cancer.

[72] Houvast R.D., Badr N., March T., de Muynck L., Sier V.Q., Schomann T., Bhairosingh S., Baart V.M., Peeters J., van Westen G.J.P. (2024). Preclinical evaluation of EpCAM-binding designed ankyrin repeat proteins (DARPins) as targeting moieties for bimodal near-infrared fluorescence and photoacoustic imaging of cancer. Eur. J. Nucl. Med. Mol. Imaging.

[73] Boogerd L.S.F., Boonstra M.C., Prevoo H., Handgraaf H.J.M., Kuppen P.J.K., van de Velde C.J.H., Fish A., Cordfunke R.A., Valentijn A., Terwisscha van Scheltinga A.G. (2019). Fluorescence-guided tumor detection with a novel anti-EpCAM targeted antibody fragment: Preclinical validation. Surg. Oncol..

[74] Bui T.M., Wiesolek H.L., Sumagin R. (2020). ICAM-1: A master regulator of cellular responses in inflammation, injury resolution, and tumorigenesis. J. Leukoc. Biol..

[75] Chen S., Pan S., Wu H., Zhou J., Huang Y., Wang S., Liu A. (2020). ICAM1 Regulates the Development of Gastric Cancer and May Be a Potential Biomarker for the Early Diagnosis and Prognosis of Gastric Cancer. Cancer Manag. Res..

[76] Maruo Y., Gochi A., Kaihara A., Shimamura H., Yamada T., Tanaka N., Orita K. (2002). ICAM-1 expression and the soluble ICAM-1 level for evaluating the metastatic potential of gastric cancer. Int. J. Cancer.

[77] Wang X., Yuan L., Guan X., Yao S., Zhu B., Liu C., Yang T., Guo P., Qin J.-J., Cheng X.-D. (2022). A novel antibody drug conjugateengineered for chromosome instable gastric cancer. Cancer Res..

[78] Wang X.Y., Chen S.H., Zhang Y.N., Xu C.F. (2018). Olfactomedin-4 in digestive diseases: A mini-review. World J. Gastroenterol..

[79] Jang B.G., Lee B.L., Kim W.H. (2015). Olfactomedin-related proteins 4 (OLFM4) expression is involved in early gastric carcinogenesis and of prognostic significance in advanced gastric cancer. Virchows Arch..

[80] Luo Z., Zhang Q., Zhao Z., Li B., Chen J., Wang Y. (2011). OLFM4 is associated with lymph node metastasis and poor prognosis in patients with gastric cancer. J. Cancer Res. Clin. Oncol..

[81] Liu R.H., Yang M.H., Xiang H., Bao L.M., Yang H.A., Yue L.W., Jiang X., Ang N., Wu L.Y., Huang Y. (2012). Depletion of OLFM4 gene inhibits cell growth and increases sensitization to hydrogen peroxide and tumor necrosis factor-alpha induced-apoptosis in gastric cancer cells. J. Biomed. Sci..

[82] Zhao J., Shu P., Duan F., Wang X., Min L., Shen Z., Ruan Y., Qin J., Sun Y., Qin X. (2016). Loss of OLFM4 promotes tumor migration through inducing interleukin-8 expression and predicts lymph node metastasis in early gastric cancer. Oncogenesis.

[83] Wang Y., Zhang L., Yang Y., Lu S., Chen H. (2021). Progress of Gastric Cancer Surgery in the era of Precision Medicine. Int. J. Biol. Sci..

[84] Zhao C., Rong Z., Ding J., Wang L., Wang B., Ding L., Meng L., Meng X., Wang F., Yang Z. (2022). Targeting Claudin 18.2 Using a Highly Specific Antibody Enables Cancer Diagnosis and Guided Surgery. Mol. Pharm..

[85] Pinho S.S., Reis C.A. (2015). Glycosylation in cancer: Mechanisms and clinical implications. Nat. Rev. Cancer.

[86] Munkley J., Elliott D.J. (2016). Hallmarks of glycosylation in cancer. Oncotarget.

[87] Houvast R.D., Baart V.M., Bhairosingh S.S., Cordfunke R.A., Chua J.X., Vankemmelbeke M., Parsons T., Kuppen P.J.K., Durrant L.G., Vahrmeijer A.L. (2020). Glycan-Based Near-infrared Fluorescent (NIRF) Imaging of Gastrointestinal Tumors: A Preclinical Proof-of-Concept In Vivo Study. Mol. Imaging Biol..

[88] Hanahan D. (2022). Hallmarks of Cancer: New Dimensions. Cancer Discov..

[89] Lambert A.W., Weinberg R.A. (2021). Linking EMT programmes to normal and neoplastic epithelial stem cells. Nat. Rev. Cancer.

[90] Liu W.F., Ji S.R., Sun J.J., Zhang Y., Liu Z.Y., Liang A.B., Zeng H.Z. (2012). CD146 expression correlates with epithelial-mesenchymal transition markers and a poor prognosis in gastric cancer. Int. J. Mol. Sci..

[91] Wang P., Qu Y., Li C., Yin L., Shen C., Chen W., Yang S., Bian X., Fang D. (2015). Bio-functionalized dense-silica nanoparticles for MR/NIRF imaging of CD146 in gastric cancer. Int. J. Nanomed..

[92] Dufresne M., Seva C., Fourmy D. (2006). Cholecystokinin and gastrin receptors. Physiol. Rev..

[93] Wayua C., Low P.S. (2014). Evaluation of a cholecystokinin 2 receptor-targeted near-infrared dye for fluorescence-guided surgery of cancer. Mol. Pharm..

[94] Mohaghegh S., Tarighatnia A., Omidi Y., Barar J., Aghanejad A., Adibkia K. (2022). Multifunctional magnetic nanoparticles for MRI-guided co-delivery of erlotinib and L-asparaginase to ovarian cancer. J. Microencapsul..

[95] Tarighatnia A., Fouladi M.R., Tohidkia M.R., Johal G., Nader N.D., Aghanejad A., Ghadiri H. (2021). Engineering and quantification of bismuth nanoparticles as targeted contrast agent for computed tomography imaging in cellular and animal models. J. Drug Deliv. Sci. Technol..

[96] Yang Z., Wang C., Du S., Ma Q., Wang W., Liu C., Zhan Y., Zhan W. (2024). Folic acid-mediated hollow Mn3O4 nanocomposites for in vivo MRI/FLI monitoring the metastasis of gastric cancer. Biomed. Eng. Online.

[97] D’Antongiovanni V., Martinelli S., Richter S., Canu L., Guasti D., Mello T., Romagnoli P., Pacak K., Eisenhofer G., Mannelli M. (2017). The microenvironment induces collective migration in SDHB-silenced mouse pheochromocytoma spheroids. Endocr. Relat. Cancer.

[98] Martinelli S., Amore F., Canu L., Maggi M., Rapizzi E. (2023). Tumour microenvironment in pheochromocytoma and paraganglioma. Front. Endocrinol..

[99] Dong Y., Hu K., Zhang J., Zhu M., Liu M., Yuan Y., Sun X., Xu Z., Li S., Zhu Y. (2024). ScRNA-seq of gastric cancer tissues reveals differences in the immune microenvironment of primary tumors and metastases. Oncogene.

[100] Liang S., Lin T., Ding J., Pan Y., Dang D., Guo C., Zhi M., Zhao P., Sun L., Hong L. (2006). Screening and identification of vascular-endothelial-cell-specific binding peptide in gastric cancer. J. Mol. Med..

[101] Liang S., Ding J., Hui X., Lv Y., Liu Y., Wu K., Fan D. (2012). Diagnostic and Therapeutic Gastric Cancer Vascular Specific Binding Peptide GEBP11 Isotope Probe.

[102] Tian Z., Liang S., Zhou X., Luo H., Tian M., Zhang X., Guo C., Zhang J. (2022). Near-infrared-dye labeled tumor vascular-targeted dimer GEBP11 peptide for image-guided surgery in gastric cancer. Front. Oncol..

[103] Cai W., Shin D.W., Chen K., Gheysens O., Cao Q., Wang S.X., Gambhir S.S., Chen X. (2006). Peptide-labeled near-infrared quantum dots for imaging tumor vasculature in living subjects. Nano Lett..

[104] Su T., Wang Y., Wang J., Han D., Ma S., Cao J., Li X., Zhang R., Qiao H., Liang J. (2016). In Vivo Magnetic Resonance and Fluorescence Dual-Modality Imaging of Tumor Angiogenesis in Rats Using GEBP11 Peptide Targeted Magnetic Nanoparticles. J. Biomed. Nanotechnol..

[105] Sikkenk D.J., Sterkenburg A.J., Schmidt I., Gorpas D., Nagengast W.B., Consten E.C.J. (2023). Detection of Tumour-Targeted IRDye800CW Tracer with Commercially Available Laparoscopic Surgical Systems. Diagnostics.

[106] Lamberts L.E., Koch M., de Jong J.S., Adams A.L.L., Glatz J., Kranendonk M.E.G., Terwisscha van Scheltinga A.G.T., Jansen L., de Vries J., Lub-de Hooge M.N. (2017). Tumor-Specific Uptake of Fluorescent Bevacizumab-IRDye800CW Microdosing in Patients with Primary Breast Cancer: A Phase I Feasibility Study. Clin. Cancer Res..

[107] Harlaar N.J., Koller M., de Jongh S.J., van Leeuwen B.L., Hemmer P.H., Kruijff S., van Ginkel R.J., Been L.B., de Jong J.S., Kats-Ugurlu G. (2016). Molecular fluorescence-guided surgery of peritoneal carcinomatosis of colorectal origin: A single-centre feasibility study. Lancet Gastroenterol. Hepatol..

[108] Ogawa S., Kubo H., Murayama Y., Kubota T., Yubakami M., Matsumoto T., Ohashi T., Okamoto K., Kuriki Y., Hanaoka K. (2021). Matrix metalloprotease-14 is a target enzyme for detecting peritoneal metastasis in gastric cancer. Photodiagnosis Photodyn. Ther..

[109] Yim J.J., Tholen M., Klaassen A., Sorger J., Bogyo M. (2018). Optimization of a Protease Activated Probe for Optical Surgical Navigation. Mol. Pharm..

[110] Martinez-Outschoorn U.E., Lin Z., Trimmer C., Flomenberg N., Wang C., Pavlides S., Pestell R.G., Howell A., Sotgia F., Lisanti M.P. (2011). Cancer cells metabolically “fertilize” the tumor microenvironment with hydrogen peroxide, driving the Warburg effect: Implications for PET imaging of human tumors. Cell Cycle.

[111] Martinelli S., Riverso M., Mello T., Amore F., Parri M., Simeone I., Mannelli M., Maggi M., Rapizzi E. (2022). SDHB and SDHD silenced pheochromocytoma spheroids respond differently to tumour microenvironment and their aggressiveness is inhibited by impairing stroma metabolism. Mol. Cell. Endocrinol..

[112] Li P., Zhang H., Chen T., Zhou Y., Yang J., Zhou J. (2024). Cancer-associated fibroblasts promote proliferation, angiogenesis, metastasis and immunosuppression in gastric cancer. Matrix Biol..

[113] Mukkamala R., Lindeman S.D., Kragness K.A., Shahriar I., Srinivasarao M., Low P.S. (2022). Design and characterization of fibroblast activation protein targeted pan-cancer imaging agent for fluorescence-guided surgery of solid tumors. J. Mater. Chem. B.

[114] Yang N., Huang Y., Wang X., Wang D., Yao D., Ren G. (2024). Fibronectin-Targeting Dual-Modal MR/NIRF Imaging Contrast Agents for Diagnosis of Gastric Cancer and Peritoneal Metastasis. Bioconjug. Chem..

[115] Han Z., Lu Z.R. (2017). Targeting Fibronectin for Cancer Imaging and Therapy. J. Mater. Chem. B.

[116] Guo W., Ren Y., Chen Z., Shen G., Lu Y., Zhou H., Li Z., Li Z., Lu X., Li G. (2023). Targeted Magnetic Resonance Imaging/Near-Infrared Dual-Modal Imaging and Ferroptosis/Starvation Therapy of Gastric Cancer with Peritoneal Metastasis. Adv. Funct. Mater..

[117] Zhang P., Tong Y., Huang X., Chen Y., Li Y., Luan D., Li J., Wang C., Li P., Du L. (2023). The Dual-Response–Single-Amplification Fluorescent Nanomachine for Tumor Imaging and Gastric Cancer Diagnosis. ACS Nano.

[118] Ma Q., Xiao J., Qiu Z., Huang H., Yan D., You Y., Wang L., Guo X. (2024). Indocyanine Green-Loaded Mesoporous Silica Nanocomposite for Breast Cancer Imaging. ACS Appl. Nano Mater..

[119] Zhao P., Xu Q., Tao J., Jin Z., Pan Y., Yu C., Yu Z. (2018). Near infrared quantum dots in biomedical applications: Current status and future perspective. Wiley Interdiscip. Rev. Nanomed. Nanobiotechnol..

[120] Yu G.-T., Luo M.-Y., Li H., Chen S., Huang B., Sun Z.-J., Cui R., Zhang M. (2019). Molecular Targeting Nanoprobes with Non-Overlap Emission in the Second Near-Infrared Window for in Vivo Two-Color Colocalization of Immune Cells. ACS Nano.

[121] Pons T., Bouccara S., Loriette V., Lequeux N., Pezet S., Fragola A. (2019). In Vivo Imaging of Single Tumor Cells in Fast-Flowing Bloodstream Using Near-Infrared Quantum Dots and Time-Gated Imaging. ACS Nano.

[122] Hamidu A., Pitt W.G., Husseini G.A. (2023). Recent Breakthroughs in Using Quantum Dots for Cancer Imaging and Drug Delivery Purposes. Nanomaterials.

[123] Gil H.M., Price T.W., Chelani K., Bouillard J.-S.G., Calaminus S.D.J., Stasiuk G.J. (2021). NIR-quantum dots in biomedical imaging and their future. iScience.

[124] Zhang Y.P., Sun P., Zhang X.R., Yang W.L. (2012). In vitro gastric cancer cell imaging using near-infrared quantum dot-conjugated CC49. Oncol. Lett..

[125] He Y., Xu H., Chen C., Peng J., Tang H., Zhang Z., Li Y., Pang D. (2011). In situ spectral imaging of marker proteins in gastric cancer with near-infrared and visible quantum dots probes. Talanta.

[126] Cao Y., Chen Z., Li X., Li Z., Lin G., Liu T., Wu Y. (2022). Dual-color quantum dot-loaded nanoparticles based lateral flow biosensor for the simultaneous detection of gastric cancer markers in a single test line. Anal. Chim. Acta.

[127] Zhang D., Wang H., Chen C., Lu G., Yin Y., Ren M., Huang J. (2024). Preparation and identification of a fluorescent probe with CsPbBr(3)perovskite quantum dots and CD44v6 specific peptide for gastric cancer imaging. Nanotechnology.

[128] Warburg O., Wind F., Negelein E. (1927). THE METABOLISM OF TUMORS IN THE BODY. J. Gen. Physiol..

[129] Kim W.H., Lee J., Jung D.-W., Williams D.R. (2012). Visualizing Sweetness: Increasingly Diverse Applications for Fluorescent-Tagged Glucose Bioprobes and Their Recent Structural Modifications. Sensors.

[130] Yaylali O., Kiraç F.S., Yüksel D. (2016). The role of 18F-FDG PET-CT in the detection of unknown primary malignancy: A retrospective study. Turk. J. Med. Sci..

[131] Zhang M., Zhang Z., Blessington D., Li H., Busch T.M., Madrak V., Miles J., Chance B., Glickson J.D., Zheng G. (2003). Pyropheophorbide 2-deoxyglucosamide: A new photosensitizer targeting glucose transporters. Bioconjug. Chem..

[132] Cheng Z., Levi J., Xiong Z., Gheysens O., Keren S., Chen X., Gambhir S.S. (2006). Near-infrared fluorescent deoxyglucose analogue for tumor optical imaging in cell culture and living mice. Bioconjug. Chem..

[133] Kovar J.L., Volcheck W., Sevick-Muraca E., Simpson M.A., Olive D.M. (2009). Characterization and performance of a near-infrared 2-deoxyglucose optical imaging agent for mouse cancer models. Anal. Biochem..

[134] Vendrell M., Samanta A., Yun S.W., Chang Y.T. (2011). Synthesis and characterization of a cell-permeable near-infrared fluorescent deoxyglucose analogue for cancer cell imaging. Org. Biomol. Chem..

[135] Guo J., Du C., Shan L., Zhu H., Xue B., Qian Z., Achilefu S., Gu Y. (2012). Comparison of near-infrared fluorescent deoxyglucose probes with different dyes for tumor diagnosis in vivo. Contrast Media Mol. Imaging.

[136] Sun W., Fan J., Hu C., Cao J., Zhang H., Xiong X., Wang J., Cui S., Sun S., Peng X. (2013). A two-photon fluorescent probe with near-infrared emission for hydrogen sulfide imaging in biosystems. Chem. Commun..

[137] Fang Y., Zhang W., Zhu M., Chen S., Liu X., Lu W., Zhang X. (2017). Characterization of a near-infrared fluorescent DCPO-tagged glucose analogue for cancer cell imaging. J. Photochem. Photobiol. B.

[138] Zhao N., Zhang C., Zhao Y., Bai B., An J., Zhang H., Wu J.B., Shi C. (2016). Optical imaging of gastric cancer with near-infrared heptamethine carbocyanine fluorescence dyes. Oncotarget.

[139] Yang X., Shi C., Tong R., Qian W., Zhau H.E., Wang R., Zhu G., Cheng J., Yang V.W., Cheng T. (2010). Near IR heptamethine cyanine dye-mediated cancer imaging. Clin. Cancer Res..

[140] Bogdanov A., Chubenko V., Volkov N., Moiseenko F., Moiseyenko V. (2022). Tumor acidity: From hallmark of cancer to target of treatment. Front. Oncol..

[141] Piasentin N., Milotti E., Chignola R. (2020). The control of acidity in tumor cells: A biophysical model. Sci. Rep..

[142] Voskuil F.J., Steinkamp P.J., Zhao T., van der Vegt B., Koller M., Doff J.J., Jayalakshmi Y., Hartung J.P., Gao J., Sumer B.D. (2020). Exploiting metabolic acidosis in solid cancers using a tumor-agnostic pH-activatable nanoprobe for fluorescence-guided surgery. Nat. Commun..

[143] Zhang C.H., Cai K., Zhang P.G., Wu Z., Ma M., Chen B. (2022). pH-Responsive DNA nanoassembly for detection and combined therapy of tumor. Biosens. Bioelectron..

[144] Abate M., Walch H., Arora K., Vanderbilt C.M., Fei T., Drebin H., Shimada S., Maio A., Kemel Y., Stadler Z.K. (2023). Unique Genomic Alterations and Microbial Profiles Identified in Patients with Gastric Cancer of African, European, and Asian Ancestry: A Novel Path for Precision Oncology. Ann. Surg..

[145] Chen C., Shen J., Du Y., Shi X., Niu Y., Jin G., Liu Y., Shi Y., Lyu J., Lin L. (2022). Characteristics of gut microbiota in patients with gastric cancer by surgery, chemotherapy and lymph node metastasis. Clin. Transl. Oncol..

[146] Yang H.J. (2023). Gastric Cancer and Gastric Microbiome. Korean J. Gastroenterol..

[147] Zeng R., Gou H., Lau H.C.H., Yu J. (2024). Stomach microbiota in gastric cancer development and clinical implications. Gut.

[148] Chattopadhyay I., Gundamaraju R., Rajeev A. (2023). Diversification and deleterious role of microbiome in gastric cancer. Cancer Rep..

[149] Bali P., Coker J., Lozano-Pope I., Zengler K., Obonyo M. (2021). Microbiome Signatures in a Fast- and Slow-Progressing Gastric Cancer Murine Model and Their Contribution to Gastric Carcinogenesis. Microorganisms.

[150] Chen J., Nie S., Qiu X., Zheng S., Ni C., Yuan Y., Gong Y. (2023). Leveraging existing 16S rRNA microbial data to identify diagnostic biomarker in Chinese patients with gastric cancer: A systematic meta-analysis. mSystems.

[151] Sarhadi V., Mathew B., Kokkola A., Karla T., Tikkanen M., Rautelin H., Lahti L., Puolakkainen P., Knuutila S. (2021). Gut microbiota of patients with different subtypes of gastric cancer and gastrointestinal stromal tumors. Gut Pathog..

[152] Wang S., Liu P., Yu J., Liu T. (2024). Multi-omics analysis revealed the regulation mode of intratumor microorganisms and microbial signatures in gastrointestinal cancer. Carcinogenesis.

[153] Sam A. (2022). A Novel Microbiome Signature in Gastric Cancer. Ann. Surg..

[154] Wang J., Wang Y., Li Z., Gao X., Huang D. (2021). Global Analysis of Microbiota Signatures in Four Major Types of Gastrointestinal Cancer. Front. Oncol..

